# Thigh muscle volume change is associated with longitudinal structural and functional knee osteoarthritis progression - Data from the osteoarthritis initiative

**DOI:** 10.1016/j.ocarto.2026.100858

**Published:** 2026-08-13

**Authors:** Sevtap Tugce Ulas, Felix Liu, Gabby B. Joseph, Sharmila Majumdar, Gabbie Hoyer, Michael C. Nevitt, Charles E. McCulloch, Nancy E. Lane, Thomas M. Link, Alexandra S. Gersing

**Affiliations:** a Department of Radiology and Biomedical Imaging, University of California, San Francisco, CA, USA; b Department of Radiology, Charité – Universitätsmedizin Berlin, Campus Mitte, Humboldt – Universität zu Berlin, Freie Universität Berlin, Berlin, Germany; c Department of Epidemiology and Biostatistics, University of California, San Francisco, CA, USA; d Department of Medicine, University of California, Davis, CA, USA

**Keywords:** Muscles, Osteoarthritis, MRI, Deep learning

## Abstract

**Objective:**

To investigate the association of change in thigh muscle volume (TMV) with structural knee changes and changes in knee pain and functional limitation over 48 months.

**Design:**

Deep learning-based automated segmentations were used to quantify magnetic resonance imaging-derived TMV at baseline and 48 months in 1715 participants from the Osteoarthritis Initiative (mean age 60.0 years; 54.3% women). Participants were categorized by quartile of percentage TMV change over 48 months into muscle loss (Q1: −29.8 to < -7.2%,n = 420), stable muscle (Q2+Q3: −7.2 to <2.4%,n = 864) and muscle gain group (Q4: 2.4–23.7%,n = 431). Structural knee changes were assessed using the Whole-Organ Magnetic Resonance Imaging Score (WORMS), changes in knee pain and functional limitations using the Western Ontario and McMaster Universities Osteoarthritis Index (WOMAC) and physical function with the Repeated Chair Stand Test (CST). Associations between TMV changes, WORMS, and functional outcomes were analyzed using multivariable and ordinal regression models.

**Results:**

Over 48 months, muscle gain was significantly (p < 0.05) associated with less worsening of bone marrow edema-like lesions (β = −0.29 [95% confidence interval: −0.47, −0.10]), reduced worsening of ligamentous damage (β = −0.12 [-0.22, −0.02]), and less increase in WOMAC pain (β = −0.36 [-0.68, −0.04]) compared to the stable muscle group. Muscle loss was associated with greater tendon damage (β = 0.08 [0.01, 0.15]) and greater declines in CST (β = −0.58 [-1.08, −0.08]). No significant associations were observed between the muscle groups for cartilage and menisci WORMS progression or for physical function (p > 0.05).

**Conclusions:**

Thigh muscle gain was associated with less progression in ligamentous damage and bone marrow edema-like lesions and pain, whereas muscle loss was associated with greater tendon progression and decline in physical performance.

## 1 Introduction

Knee osteoarthritis (KOA) is one of the leading causes of chronic pain, functional limitations and disability worldwide, affecting millions of individuals and placing a significant burden on healthcare systems [[1], [2], [3]]. KOA impacts patients’ quality of life and daily activities, often resulting in reduced mobility, difficulty with weight-bearing tasks, and reliance on healthcare resources [4]. Pain and functional impairment are hallmark features of KOA, often worsening due to progressive structural damage in the knee joint [5]. Structural changes, including cartilage degeneration, changes in subchondral bone and meniscal damage are central to the pathophysiology of KOA and are closely associated with worsening disease severity [6]. Advanced imaging techniques, such as MRI, are critical for identifying structural changes and quantifying disease progression. Scoring systems such as the Whole-Organ Magnetic Resonance Imaging Score (WORMS) provide detailed assessments of structural KOA severity by evaluating various joint compartments, including cartilage, bone marrow, ligaments, tendons and menisci [7]. These imaging-based assessments are critical for understanding KOA progression, as structural damage often correlates with increased pain and functional decline.

Muscle plays a crucial role in maintaining joint stability, mobility, and biomechanical balance, serving as a key component of the musculoskeletal system [8,9]. The integrity of muscle function is essential for preserving joint health, as it ensures optimal load distribution and stabilization during movement. Sarcopenia, characterized by a decrease in muscle mass, strength, and function, has been identified as a major risk factor for functional decline and disease progression in KOA patients [10]. Muscle loss can lead to altered joint biomechanics and increased cartilage stress, resulting in accelerated structural degeneration [11]. Recent evidence further emphasizes the important role of muscle impairment in KOA. A recent systematic review showed consistent neuromuscular deficits associated with pain and functional limitations [12], while reduced quadriceps strength was associated with worse WOMAC scores and impaired functional performance in patients with hip or knee osteoarthritis [13]. Previous studies have established the link between muscle weakness and joint instability [11]. For example, Mohajer et al. reported that longitudinal changes in quadriceps cross-sectional area and intramuscular adipose tissue, derived from single-slice MRI measurements, were associated with symptom progression and risk of knee replacement [14]. Most MRI studies investigating muscle in KOA have relied on single-slice cross-sectional measurements. However, single-slice measurements may not fully capture the complex three-dimensional distribution of muscle tissue and may be limited in representing global muscle morphology [15]. Therefore, despite existing evidence linking muscle characteristics to KOA outcomes, the association between longitudinal changes in volumetric muscle measures and structural joint damage over a long term and in a large cohort, as well as their impact on functional outcomes, remains less explored. In particular, the potential value of fully automated, deep learning-based assessments of thigh muscle volume (TMV) for providing additional or more robust information with structural disease progression has not yet been established. Volumetric assessment may capture muscle morphology more comprehensively than previously used single-slice or area-based approaches [14]. However, a direct comparison is beyond the scope of our study.

Functional limitations in KOA are frequently assessed using the Western Ontario and McMaster Universities Osteoarthritis Index (WOMAC), a validated, widely used tool designed to quantify pain, stiffness, and physical function in individuals with knee or hip OA [16]. Higher WOMAC scores reflect greater levels of pain, stiffness, and functional limitations, which are typically associated with more severe disease progression in KOA [17]. However, the association between changes in volumetric muscle measures and functional outcomes, as measured by WOMAC, remains unclear [18].

Given the critical role of muscle in KOA structural and functional outcomes, this study aimed to investigate the longitudinal association between changes in deep learning-based thigh muscle volume, structural progression of KOA, and functional impairment over 48 months. We hypothesized that muscle loss would be associated with greater progression of degenerative changes (WORMS score increase) and worse functional outcomes relative to stable muscle, and that muscle gain would be associated with slowed progression of degenerative changes (stable WORMS score).

## 2 Method

### 2.1 Database and participants

The study population was derived from the Osteoarthritis Initiative (OAI) (http://www.oai.ucsf.edu), a prospective, multicenter cohort study designed to include individuals who were either healthy with or without risk factors for knee OA or had symptomatic knee OA. The analytic cohort comprised a mixed OAI population, including participants with mild established knee OA as well as individuals at increased risk of developing knee OA, and therefore was not restricted to participants with established symptomatic or radiographic disease.

The dataset included the WOMAC questionnaire [16] with three subcategories: pain (0–20 range), stiffness (0–8 range), and functional limitation (0–68 range). Physical performance and strength were assessed by performing the Repeated Chair Stand Test (CST) (ability to complete 5 CST - yes/no) at baseline and at the 48-month follow-up.

**Fig. 1:**
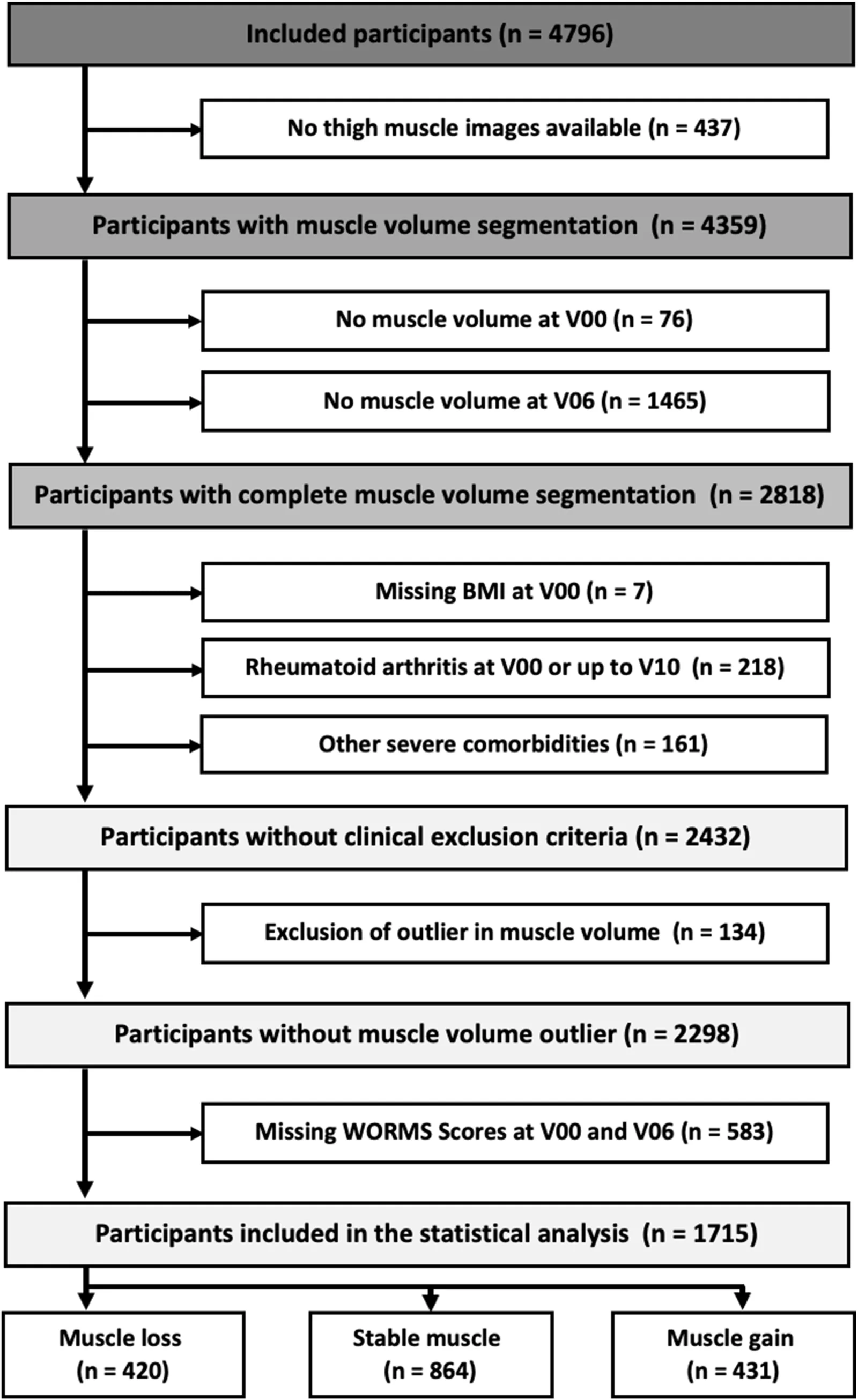
Flowchart of study inclusion. V00 = baseline, V06 = follow-up after 48 months, BMI = body mass index, WORMS = Whole-Organ Magnetic Resonance Imaging Score.

MRI-derived TMV data were available for 2818 participants at baseline and 48-month follow-up. Participants were excluded if baseline body mass index (BMI) data were missing (n = 7), if they had rheumatoid arthritis at baseline or developed rheumatoid arthritis during the study period (n = 218), or if severe comorbidities (cancer or stroke) were present (n = 161). Participants with inflammatory arthritis were excluded irrespective of the timing of diagnosis to ensure that the study population represented degenerative rather than inflammatory joint disease. In total, 1715 participants were included in the analysis (see Fig. 1).

### 2.2 Ethical approval

Written informed consent was obtained from all participants, and the study adhered to the regulations outlined in the Health Insurance Portability and Accountability Act (HIPAA). Ethical approval was granted by the institutional review boards of all participating centers.

### 2.3 MR imaging

MRI scans of the right knee and thigh were conducted at baseline and at the 48-month follow-up according to the standardized OAI imaging protocol using four identical 3T scanners (Siemens Magnetom Trio; Siemens Healthcare, Erlangen, Germany) equipped with transmit-receive coils (USA Instruments, Aurora, OH) across four imaging centers. TMV was assessed using T1-weighted axial sequences with the following imaging parameters: repetition time (TR)/echo time (TE) 600/10 ms, slice thickness of 5 mm, and a craniocaudal coverage of approximately 7.5 cm, beginning 10 cm proximal to the right femoral epiphysis [19,20]. Further details regarding the analyzed MRI sequences can be found in the OAI protocol [21].

### 2.4 Deep learning-based automated muscle analysis

At both timepoints, 15 axial slices of the right thigh were acquired, with the central 7 slices selected as the primary unit of observation for analysis to minimize the effect of image quality degradation in the edge slices. The fully automated artificial intelligence-enabled MR muscle segmentation pipeline consisted of a fine-tuned YOLO (You Only Look Once) model for bounding box detection and a fine-tuned box-prompted SAM (Segment Anything Model) segmentation model for the quadriceps, hamstrings, adductors, gracilis, and sartorius muscles [22]. The pipeline was trained and validated using manual segmentations on all slices of 109 thigh MRI volumes from three musculoskeletal radiologists, and tested on a hold out cohort with manual central slice segmentations on 246 from two musculoskeletal radiologists. In the hold out test cohort, the mean Dice similarity coefficient was 0.96 for both the quadriceps and hamstrings. The coefficients of variation were 0.3% for the quadriceps and 2.5% for the hamstrings, with mean biases of −1.7% and +1.2%, respectively, compared with the manual reference standard [22]. Muscle sub-volumes (in cm^3^) for all included muscle groups were calculated at baseline and at the 48-month follow-up to quantify changes in muscle volume over time.

### 2.5 Structural knee joint analysis

All knee MRI datasets at baseline and at 48-months follow-up were separately anonymized before being evaluated. OA-related features were assessed using the modified semiquantitative WORMS grading system [7,23,24]. WORMS grading was performed using the same standardized reading protocol and readers as previously described [25], for which good-to-excellent intra- and inter-reader reproducibility has been reported. Meniscal defects in the medial and lateral meniscus (anterior, body, posterior) were graded on a scale of 0–4: 0 = intact, 1 = intrasubstance signal abnormalities, 2 = nondisplaced tear, 3 = displaced or complex tear, 4 = maceration. Ligament abnormalities (anterior/posterior cruciate ligaments, medial/lateral collateral ligaments) and tendon pathologies (patellar and popliteal tendons) were graded on a 0–4 scale: 0 = no pathology, 1 = signal abnormalities around the tendon/ligament, 2 = signal abnormalities within the tendon/ligament, 3 = partial tear, and 4 = complete tear. Cartilage defects were graded on a 0–6 scale, and bone marrow edema-like lesions (BMELL) on a 0–3 scale across six distinct knee regions: patella, trochlea, medial/lateral femur, and medial/lateral tibia. Tissue-specific WORMS sum scores for cartilage, meniscal abnormalities, BMELL, tendon abnormalities, and ligament abnormalities were calculated by summing the regional WORMS scores across all evaluated knee subregions. Higher scores indicate a greater overall burden of structural abnormalities within the respective tissue, with all subregions contributing equally to the corresponding tissue-specific sum score. This approach has been used previously to summarize the overall burden of tissue-specific structural abnormalities [26]. Only the right knee and right thigh were analyzed because WORMS assessments were available only for the right knee in the imaging dataset used for this study.

### 2.6 Statistical analysis

Statistical analyses were performed using R version 4.5.2 (R Core Team, 2025) using RStudio version 2025.09.2 + 418 (Posit Software PBC, Boston, MA, USA). TMV was analyzed at baseline and 48-month follow-up, with percentage change in muscle volume calculated for each participant. For the primary analyses, participants were classified into three groups based on quartiles of percentage TMV change: muscle loss (−29.8 to < −7.2%), comprising individuals in the lowest quartile (Q1); stable/minimal muscle change (−7.2% to <2.4%), including participants in the second and third quartiles (Q2–Q3); and muscle gain (2.4–23.7%), comprising individuals in the highest quartile (Q4). This categorization was chosen to compare participants with the greatest muscle loss and greatest muscle gain while using the two middle quartiles as a reference group representing relatively small changes in muscle volume. As a sensitivity analysis, percentage TMV change was additionally analyzed as a continuous variable using the same multivariable regression models. To minimize the influence of extreme values, participants with percentage TMV changes exceeding the upper bound (Q3 + 2 × interquartile range (IQR)) or falling below the lower bound (Q1 − 2 × IQR) were classified as outliers and excluded from the analyses. Baseline characteristics, including age, sex, BMI, muscle volume, mean WORMS sum scores and mean WOMAC scores, were summarized using descriptive statistics for the overall study cohort, as well as for each muscle change group individually. To assess the potential for selection bias resulting from missing WORMS scores, baseline characteristics were compared between participants with complete and missing WORMS data. Continuous variables were compared using Welch's *t*-test and categorical variables using Pearson's χ^2^ test. Standardized mean differences (SMDs) were additionally calculated to quantify the magnitude of between-group differences independent of sample size. SMDs <0.10 were considered to indicate negligible imbalance, 0.10–0.20 small imbalance, and >0.20 moderate imbalance.

Due to the large number of outcome parameters, analyses were prespecified and grouped into primary and exploratory outcomes [27,28]. Primary outcomes comprised WORMS BMELL, cartilage, and ligament score, WOMAC pain subscore, because these measures represent key structural and symptomatic features of KOA that have been consistently associated with disease progression in previous studies [27,28]. Exploratory outcomes included WORMS menisci and tendon score, WOMAC stiffness, and disability subscores and CST performance, for which associations with TMV are less well established. To investigate the hypotheses that participants with muscle loss experience greater progression in structural joint damage (evaluated by WORMS scores) and in functional outcomes (evaluated by WOMAC scores) over 48 months compared to those with muscle gain, multivariable linear regression models were conducted. These models were adjusted for sex, age, race and BMI (at baseline), as these variables were considered potential confounders based on previous literature describing differences in body composition and knee osteoarthritis across demographic groups [29]. The independent variables included changes in muscle volume/muscle group, while the outcome variables were changes in WORMS sum scores for cartilage defects, meniscal defects, ligamentous pathologies, tendon pathologies and BMELL across the six knee regions (patella, trochlea, medial femur, lateral femur, medial tibia and lateral tibia) as well as changes in the three WOMAC subscores (pain, stiffness and disability) between baseline and 48 months. Model assumptions for the linear regression analyses were assessed using standard diagnostic procedures (values are shown for the BMELL model as a representative example). Linearity was evaluated using residual-versus-fitted plots. Normality of residuals was assessed using normal Q–Q plots and the Shapiro–Wilk test (p < 0.001). Homoscedasticity was assessed using the studentized Breusch–Pagan test (BP = 13.89, p = 0.085) and Levene's test (F = 1.58, p = 0.207). Multicollinearity was evaluated using variance inflation factors (all VIFs ≤1.03), influential observations using Cook's distance and leverage values (maximum Cook's distance = 0.104; maximum leverage = 0.094), and independence of residuals using the Durbin–Watson statistic (1.98, p = 0.322). Although the Shapiro–Wilk test indicated a statistically significant deviation from normality, visual inspection of the Q–Q plots suggested only minor departures from normality, which were considered acceptable given the large sample size.

Because the primary hypotheses focused on the association between muscle gain (relative to the stable muscle group) and four prespecified primary outcomes (WORMS BMELL, cartilage, ligament, and WOMAC pain), p-values for these primary regression coefficients were additionally adjusted using the Benjamini–Hochberg false discovery rate (FDR) procedure. Exploratory outcomes were not adjusted for multiplicity and should be interpreted as hypothesis-generating. As a sensitivity analysis, all multivariable regression models evaluating WORMS outcomes were additionally adjusted for baseline physical activity (PASE score) to assess potential confounding by habitual physical activity.

To assess the association between changes in CST performance and changes in muscle volume, an ordinal regression analysis, adjusted for sex, age, race, and BMI, was conducted (independent variable: muscle group; dependent variable: change in CST, categorized into three ordered levels: deterioration (−1; able to complete the CST at baseline but unable at 48 months), stability (0; no change in completion status, i.e., able at both visits or unable at both visits), or improvement (1; unable to complete the CST at baseline but able at 48 months)).

## 3 Results

### 3.1 Participant characteristics

Participant baseline characteristics are presented in Table 1. When comparing participants with muscle gain and muscle loss to those with stable muscle, no significant differences between the groups were found regarding sex, mean age (with a SMD of −0.13 for muscle gain and SMD = 0.09 for muscle loss), and BMI at baseline (for muscle gain SMD = −0.12; for muscle loss SMD = 0.15). Of the 2298 eligible participants, 1715 (74.6%) had complete WORMS data and were included in the analysis, whereas 583 (25.4%) were excluded because of missing WORMS scores (Supplementary Table S1). Compared with included participants, those with missing WORMS data were slightly older (61.3 ± 9.3 vs. 60.0 ± 8.9 years; SMD = 0.15) and had a lower BMI (26.6 ± 5.0 vs. 28.8 ± 4.3 kg/m^2^; SMD = 0.49). Physical activity (PASE score; SMD = 0.04) was similar between groups.

**Table 1:** Baseline Characteristics. SD = standard deviation, BMI = body mass index, WORMS = Whole-Organ Magnetic Resonance Imaging Score. BMELL = bone marrow edema-like lesions. Baseline thigh muscle volume was significantly lower in the muscle gain group compared to the muscle loss group (p < 0.01) and stable muscle group (p < 0.01). No significant differences between the groups were found regarding mean age and BMI at baseline, as well as in sex, mean WOMAC subscores and mean WORMS sum scores, except for the cartilage WORMS sum score. The mean cartilage WORMS sum score at baseline was significantly lower in the muscle gain group compared to the muscle loss group (p = 0.02). The muscle loss group showed a significantly higher mean cartilage WORMS sum score compared to the stable muscle group (p = 0.04). Statistically significant results (p < 0.05) are shown in bold.

	Baseline Characteristics	All participants (n = 1715)	Muscle gain group (n = 431)	Stable muscle group (n = 864)	Muscle loss group (n = 420)
**Mean WORMS sum score**	Mean age, y (SD)	60.0 (8.9)	59.0 (8.2)	60.1 (8.9)	60.8 (9.4)
	Female sex (%)	931 (54.3)	242 (56.1)	462 (53.5)	227 (54.0)
	Mean BMI, kg/m^2^ (SD)	28.8 (4.3)	28.3 (4.1)	28.8 (4.3)	29.0 (4.3)
	Baseline muscle volume, cm^3^ (SD)	745.2 (188.6)	**702.4 (179.6)**	759.5 (183.0)	**759.9 (202.3)**
	Menisci (SD)	3.4 (3.8)	3.4 (3.9)	3.4 (3.9)	3.4 (3.8)
	Ligament (SD)	0.4 (0.9)	0.4 (1.0)	0.4 (0.9)	0.4 (1.0)
	Tendon (SD)	0.2 (0.7)	0.3 (0.8)	0.3 (0.7)	0.2 (0.6)
	Cartilage (SD)	6.9 (5.3)	**6.5 (4.9)**	6.9 (5.4)	**7.6 (5.6)**
	BMELL (SD)	2.2 (2.2)	2.2 (2.2)	2.1 (2.2)	2.4 (2.3)
**Mean WOMAC Subscores**	Pain (SD)	2.1 (2.9)	2.0 (2.7)	2.0 (2.8)	2.2 (3.1)
	Disability (SD)	6.5 (9.0)	6.2 (8.5)	6.3 (8.6)	7.0 (10.1)
	Stiffness (SD)	1.4 (1.5)	1.3 (1.5)	1.4 (1.5)	1.5 (1.5)

### 3.2 Baseline clinical and structural characteristics by muscle change group

No significant differences were observed between the groups for WOMAC scores (pain: SMD = −0.01 for muscle gain, SMD = 0.06 for muscle loss; stiffness: SMD = −0.05 for muscle gain, SMD = 0.04 for muscle loss; functional limitation: SMD = −0.01 for muscle gain, SMD = 0.07 for muscle loss) or WORMS sum scores (menisci: SMD = 0.03 for muscle gain, SMD = −0.01 for muscle loss; ligaments: SMD = 0.01 for muscle gain, SMD = −0.01 for muscle loss; tendons: SMD = 0.05 for muscle gain, SMD = −0.10 for muscle loss; cartilage: SMD = −0.06 for muscle gain and 0.13 for muscle loss). Comparing the mean cartilage WORMS sum score at baseline between the muscle gain group and muscle loss group, the muscle gain group showed a significantly lower WORMS cartilage sum score (6.5 ± SD 4.9),p = 0.02). The muscle loss group had a significantly higher WORMS cartilage sum score than the stable group (7.6 ± 5.6,p = 0.04). Baseline TMV was significantly lower in the muscle gain group (702.4 ± 179.6 cm^3^,p < 0.01) compared to the muscle loss (759.9 ± 202.3 cm^3^,p < 0.01) and stable muscle group (759.5 ± 183.0 cm^3^,p < 0.01), with no significant difference between the muscle loss and stable muscle group (p = 0.99).

### 3.3 Longitudinal changes in muscle and WORMS sum scores

**Fig. 2:**
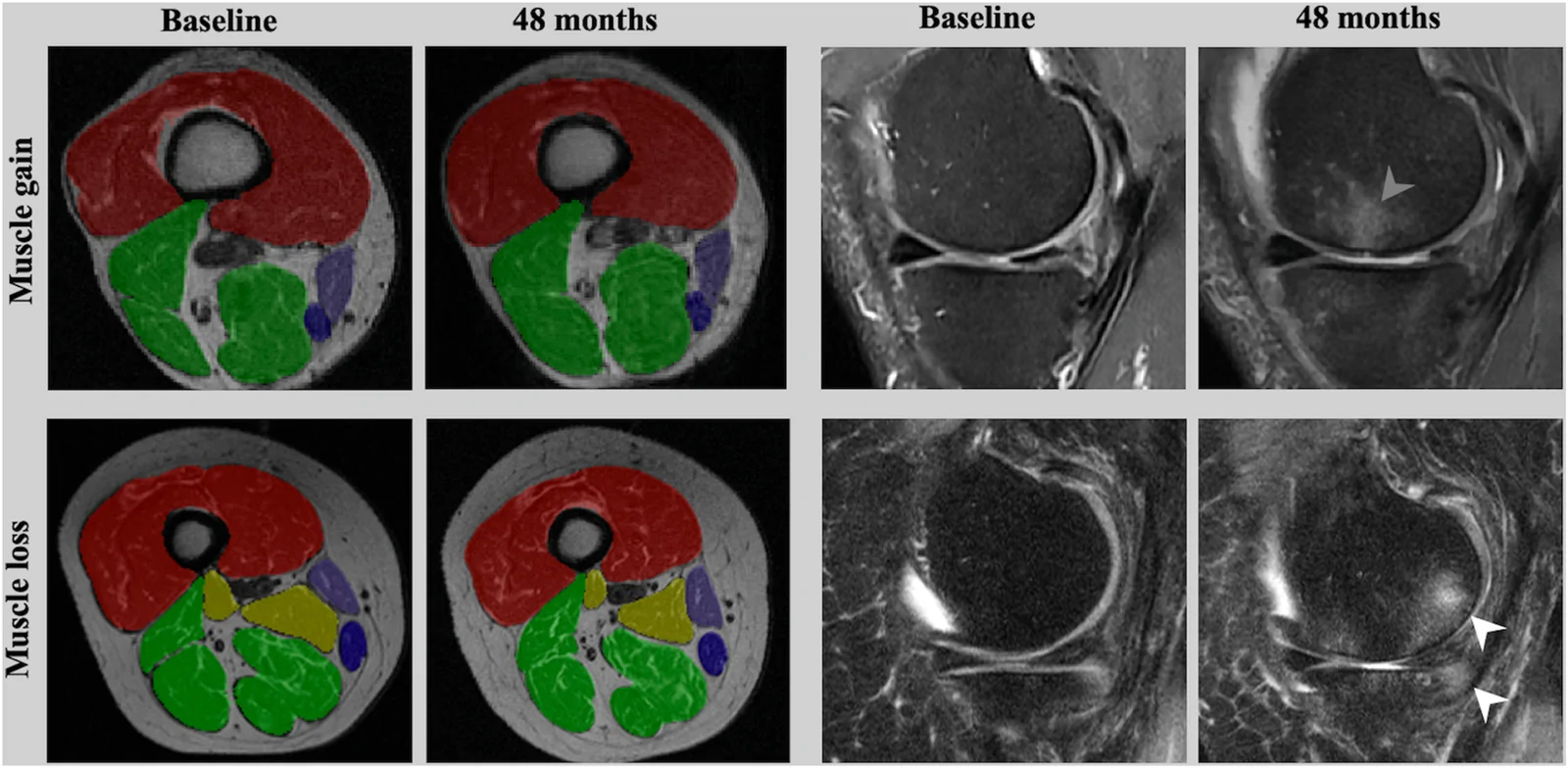
Imaging examples of changes in bone marrow edema-like lesions (BMELL). Left: Axial T1-weighted MR images of the right thigh with artificial intelligence-enabled muscle segmentation at baseline and after 48 months. Right: Sagittal intermediate-weighted fast spin-echo fat-suppression sequence of the right knee at baseline and after 48 months. The patient with muscle gain (predominantly in the quadriceps muscle, indicated in red, with an approximately 11% increase in total muscle volume over 48 months) was a 65-year-old man with mild knee pain at baseline (WOMAC pain subscale of 1) and no knee pain at 48 months (WOMAC pain subscale of 0). Despite the clinical improvement, new BMELLs developed in the medial femoral condyle (grey arrowhead), with baseline WORMS BMELL grades of 0 and 1 for medial femoral condyle at 48 months. The patient with muscle loss (predominantly in the quadriceps muscle (red) and hamstrings (green) with an approximately 14% decrease in total muscle volume over 48 months) was a 64-year-old woman with mild knee pain at baseline (WOMAC pain subscale of 1) and an increase of knee pain at 48 months (WOMAC pain subscale of 11). New BMELL developed in the medial tibial plateau and in the medial femoral condyle (white arrowheads), with baseline WORMS BMELL grades of 0 and 3 for both medial tibial plateau and medial femoral condyle at 48 months.

No significant differences were observed for adjusted mean changes in meniscus (muscle gain: 0.87 [95%CI: 0.48, 1.25], stable muscle: 0.85 [0.49, 1.22], muscle loss: 1.01 [0.62, 1.39]) or cartilage (muscle gain: 1.94 [1.38, 2.49], stable muscle: 1.98 [1.46, 2.51], muscle loss: 2.14 [1.58, 2.69] scores across the muscle groups. For BMELL scores, the muscle gain group (0.16 [−0.16, 0.48]) showed significantly lower adjusted mean changes compared to the stable muscle group (0.45 [0.14, 0.75],p = 0.03), while the comparison between muscle gain and muscle loss (0.47 [0.15, 0.80],p = 0.11) and muscle loss and stable muscle (p = 1.00) was not significant. Imaging examples of changes in BMELL are shown in Fig. 2.

**Fig. 3:**
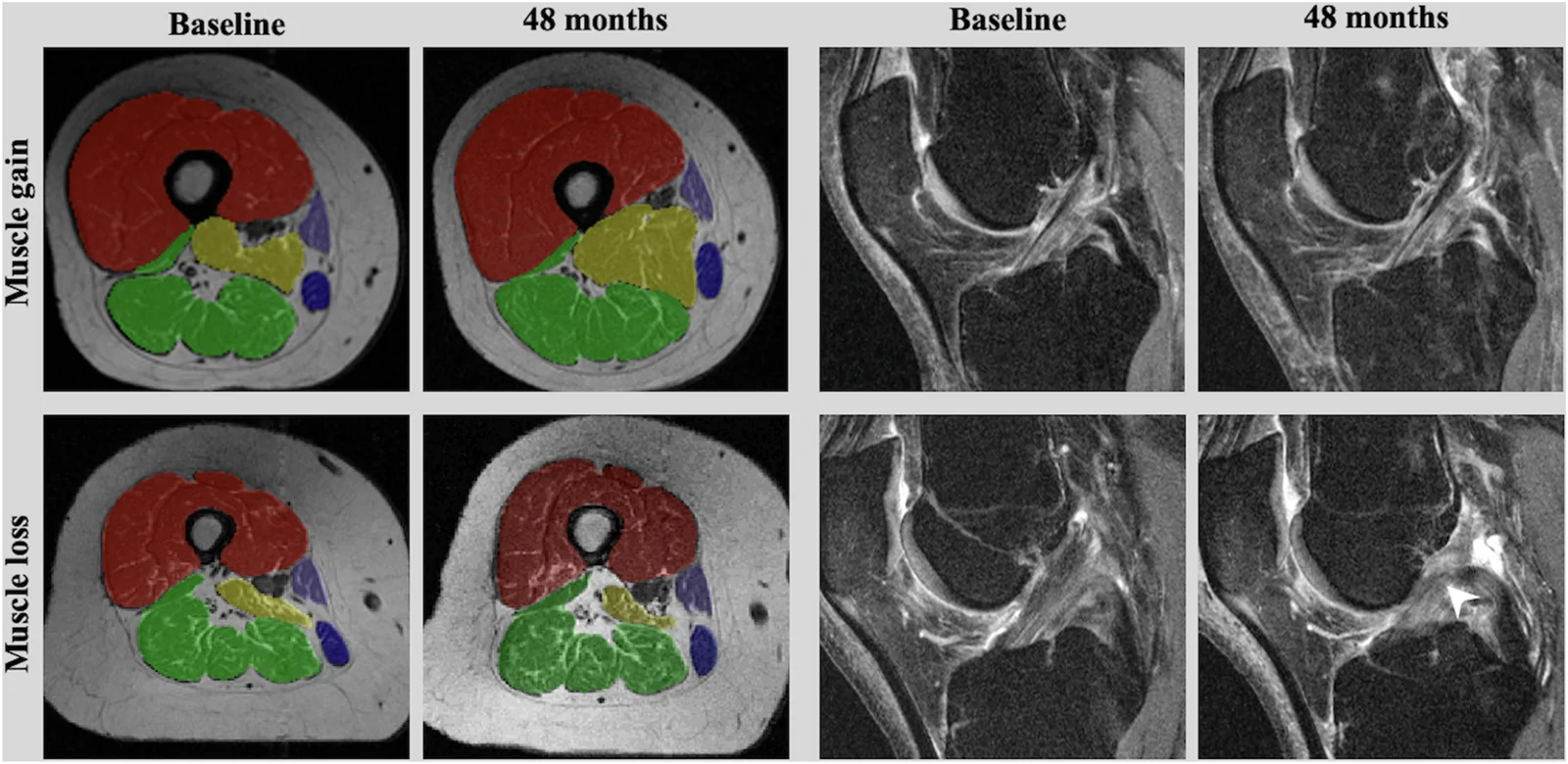
Imaging examples of changes in anterior cruciate ligament (ACL). Left: Axial T1-weighted MR images of the right thigh with artificial intelligence-enabled muscle segmentation at baseline and after 48 months. Right: Sagittal intermediate-weighted fast spin-echo fat-suppression sequence of the right knee at baseline and after 48 months. The patient with muscle gain (predominantly of the adductors (yellow) and quadriceps (red), with an approximately 6% increase in total muscle volume over 48 months) was a 53-year-old woman without knee pain at baseline and at 48 months (WOMAC pain subscores of 0) showed no change of the ACL (WORMS ACL subscore of 1 for both at baseline and at 48 months). The patient with muscle loss (predominantly of the quadriceps (red), with an approximately 9% decrease in total muscle volume) was a 62-year-old man with mild knee stiffness at baseline and at 48 months (both with a WOMAC stiffness subscore of 2) showed a new partial tear of the ACL after 48 months (white arrowhead), with baseline WORMS ACL subscore of 0 and 3 at 48 months.

**Fig. 4:**
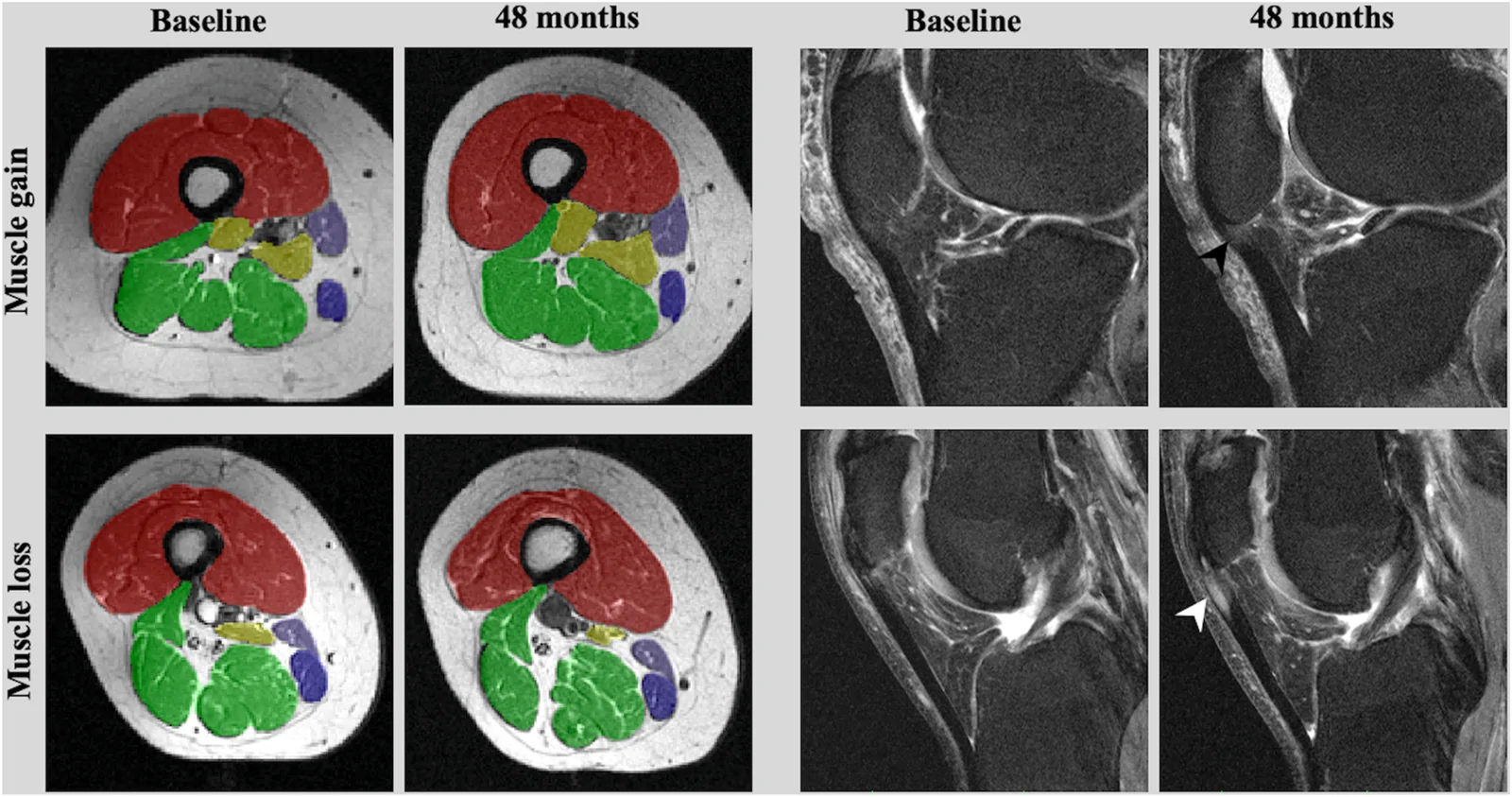
Imaging examples of changes in the patellar tendon. Left: Axial T1-weighted MR images of the right thigh with artificial intelligence-enabled muscle segmentation at baseline and after 48 months. Right: Sagittal intermediate-weighted fast spin-echo fat-suppression sequence of the right knee at baseline and after 48 months. The patient with muscle gain (predominantly of the quadriceps (red) and hamstrings (green), with an approximately 8% increase in total muscle volume over 48 months) was a 78-year-old man with a slight decrease in knee pain over 48 months (WOMAC pain subscore at baseline of 3 and after 48 months a score of 2) showed a new signal abnormalities around the patellar tendon at 48 months (black arrowhead), with a baseline WORMS patellar tendon subscore of 0 and 1 at 48 months). The patients with muscle loss (predominantly of the quadriceps (red), with an approximately 8% decrease in total muscle volume) was a 66-years-old man mild knee pain at baseline (WOMAC pain subscore of 1) and an increase of knee pain over 48 months (WOMAC pain subscore of 7 at 48 months) showed an increased tendinopathy of the patellar tendon (white arrowhead), with baseline WORMS patellar tendon subscore of 0 and 2 at 48 months.

Significant differences were found for ligament and tendon scores: for ligament score changes, the muscle gain group (0.02 [−0.15, 0.19]) showed significantly lower adjusted mean changes compared to the muscle loss group (0.14 [−0.03, 0.31],p = 0.04), while the comparison between the muscle gain and stable muscle group (0.14 [−0.02, 0.29],p = 0.08) and muscle loss and stable muscle group (p = 0.61) was not significant. Fig. 3 shows imaging examples of ligament pathologies. For tendon score changes over 48 months (Fig. 4), the muscle gain group (0.05 [−0.07, 0.16]) showed significantly smaller adjusted mean changes compared to the muscle loss group (0.15 [0.03, 0.26],p = 0.02). Comparisons between the muscle gain and stable muscle group (0.07 [−0.04, 0.18],p = 0.87) and muscle loss and stable muscle group (p = 0.12) were not significant (Table 2).

**Table 2:** Differences in mean change (Δ) in WORMS (Whole-Organ Magnetic Resonance Imaging Score) sum score over 48 months between the muscle groups adjusted for sex, age, body mass index, and race. BMELL = bone marrow edema-like lesions. Statistically significant results (p < 0.05) are shown in bold.

Change in WORMS over 48 months	Muscle gain group	Stable muscle group	Muscle loss group	P value for *muscle gain vs. muscle loss*	P value for *muscle gain vs. stable muscle*	P value for *muscle loss vs. stable muscle*
Δ mean Menisci (95% CI)	0.87 (0.48, 1.25)	0.85 (0.49, 1.22)	1.01 (0.62, 1.39)	0.50	0.65	0.75
Δ mean Ligament (95% CI)	0.02 (−0.15, 0.19)	0.14 (−0.02, 0.29)	0.14 (−0.03, 0.31)	**0.04**	0.08	1.00
Δ mean Tendon (95% CI)	0.05 (−0.07, 0.16)	0.07 (−0.04, 0.18)	0.15 (0.03, 0.26)	**0.02**	0.87	0.12
Δ mean Cartilage (95% CI)	1.94 (1.38, 2.49)	1.98 (1.46, 2.51)	2.14 (1.58, 2.69)	0.06	0.75	0.07
Δ mean BMELL (95% CI)	0.16 (−0.16, 0.48)	0.45 (0.14, 0.75)	0.47 (0.15, 0.80)	0.11	**0.03**	1.00

Multivariable regression analyses revealed significant associations between muscle group and changes in WORMS sum score over 48 months. Muscle gain was significantly associated with less WORMS progression in the ligaments (β = −0.12 [−0.22, −0.02],p = 0.01), while muscle loss was significantly associated with higher WORMS progression of the tendon score (β = 0.08 [0.01, 0.15],p = 0.02), suggesting worsening of tendon pathologies in the muscle loss group. Furthermore, muscle gain was significantly associated with less WORMS progression in BMELL (β = −0.29 [−0.47, −0.10],p = 0.002). Given the mean baseline BMELL score of 2.2 points, this corresponds to an estimated difference of approximately 13% of the average baseline BMELL burden. No significant associations were observed for the WORMS progression in cartilage (muscle gain vs. stable: β = −0.05 [−0.37, 0.27]; muscle loss vs. stable: β = 0.15 [−0.17, 0.48]) and menisci in the muscle groups (muscle gain vs. stable: β = 0.01 [−0.21, 0.23]; muscle loss vs. stable: β = 0.16 [−0.07, 0.38]) (see also Supplementary Table S2). After Benjamini–Hochberg false discovery rate adjustment for the prespecified primary outcomes, the associations between muscle gain and reduced BMELL progression (adjusted p = 0.008), ligament progression (adjusted p = 0.02), and WOMAC pain (adjusted p = 0.04) remained statistically significant. No significant association was observed for cartilage progression (adjusted p = 0.75) (see Supplementary Table S3). Sensitivity analyses using percentage TMV change as a continuous exposure variable showed associations in the same direction as the primary categorical analyses. Associations with ligament, tendon, and BMELL progression showed a statistical trend, yet did not reach the statistical level of significance (Supplementary Table S4). Additional adjustment for baseline physical activity (PASE score) did not alter the estimated associations between muscle group and changes in WORMS outcomes. Regression coefficients and statistical significance remained essentially unchanged (Supplementary Table S5). In a sensitivity analysis including participants with extreme TMV changes, the association between muscle gain and reduced ligament progression remained statistically significant, whereas the association with BMELL progression was attenuated and no longer reached statistical significance (Supplementary Table S6).

### 3.4 Longitudinal changes in muscle and clinical scores

The WOMAC pain subscore decreased slightly in all groups, with adjusted mean changes of −1.04 [−1.61, −0.48] for muscle gain, −0.69 [−1.22, −0.15] for stable muscle, and −0.68 [−1.25, −0.11] for muscle loss. Similarly, the WOMAC functional subscore showed slight reductions, with adjusted mean changes of −2.66 [−4.35, −0.96], −1.74 [−3.34, −0.14], and −1.97 [−3.66, −0.27] for muscle gain, stable muscle, and muscle loss, respectively. Changes in WOMAC stiffness subscore were minimal across all groups, with adjusted mean changes of −0.31 [−0.62, −0.01] for muscle gain, −0.26 [−0.55, 0.03] for stable muscle, and −0.25 [−0.56, 0.05] for muscle loss.

Multivariable regression analyses revealed significant association between muscle gain and the reduction in WOMAC pain score (β = −0.36 [−0.68, −0.04],p = 0.03) when compared with the stable muscle group, while no significant association was found for the muscle loss group (β = −0.004 [−0.33, 0.32]) as well as for the WOMAC stiffness (muscle gain vs. stable: β = −0.07 [−0.24, 0.11]; muscle loss vs. stable: β = −0.003 [−0.18, 0.17]) and disability subscores (muscle gain vs. stable: β = −0.88 [−1.83, 0.07]; muscle loss vs. stable: β = −0.17 [−1.12, 0.79]) (see also Supplementary Table S2).

However, no significant differences in adjusted mean CST change were observed between the muscle groups (all p > 0.05). To further evaluate functional change, an ordinal regression analysis was performed using categorized CST change (deterioration, stable, improvement) as the outcome while adjusting for age, sex, race, and BMI. In this analysis, the muscle loss group was significantly associated with a greater likelihood of functional deterioration compared with the stable muscle group (β = −0.58 [−1.08, −0.08], p = 0.02), indicating higher declines in performance over time. No significant associations were observed for the muscle gain group (β = −0.21 [−0.70, 0.29], p = 0.41).

## 4 Discussion

This study investigated the longitudinal association between changes in TMV, structural progression of KOA, and functional outcomes over 48 months in a large cohort with individuals at risk of or with mild KOA. Our findings show that changes in muscle volume are associated with progression of structural damage in the knee joint and functional impairment, especially with the progression of BMELL and WOMAC pain.

The WORMS analyses demonstrated distinct associations between muscle gain and muscle loss with structural progression over 48 months. Muscle gain was significantly associated with reduced progression of ligament abnormalities and BMELL, whereas muscle loss was significantly associated with greater progression of tendon abnormalities. These findings suggest that preservation or gain of muscle volume may be associated with reduced progression of specific structural features of KOA, while muscle loss may particularly contribute to tendon deterioration. Although the observed regression coefficients were modest in absolute terms, the association with BMELL corresponded to approximately 13% of the average baseline BMELL score. As no established minimal clinically important differences are available for individual WORMS subscores, the clinical relevance of these findings should be interpreted with caution and confirmed in future studies. Furthermore, the sensitivity analysis including participants with extreme TMV changes demonstrated that the association with ligament progression remained stable, whereas the association with BMELL progression was attenuated. This suggests that the BMELL findings may be more sensitive to extreme TMV values and should therefore be interpreted with caution. The WORMS cartilage outcome showed a statistical trend for the difference between the muscle gain and muscle loss groups, suggesting less cartilage progression in the muscle gain compared to the muscle loss group, which may indicate slowed degenerative changes in individuals with muscle gain over 4 years. Conversely, muscle loss was associated with greater progression in tendon WORMS scores, highlighting the detrimental effects of muscle decline on joint structures. These findings are consistent with prior studies linking muscle characteristics to KOA progression [14]. Our results build on previous studies by demonstrating associations between longitudinal changes in multi-slice-based thigh muscle volume and structural as well as functional outcomes in knee osteoarthritis. Although volumetric assessment may be less susceptible to slice-positioning variability than slice-slice approaches, this study did not directly compare the two methods.

The relationship between thigh muscle and KOA is likely bidirectional, as worsening KOA may impair physical activity through pain and joint dysfunction, leading to muscle loss, while muscle decline may exacerbate KOA progression through reduced joint stability and altered biomechanics [30]. The association between muscle gain and lower WOMAC pain scores may reflect a stabilizing effect on the joint, though the observational design precludes causal inference. Pain reduction is a critical outcome for KOA patients, as it not only improves physical function but also enhances overall well-being [31,32]. However, the absence of significant associations between muscle volume changes and WOMAC functional limitation and stiffness subscores indicates that muscle gain alone may not fully address all dimensions of functional impairment. Interestingly, WOMAC scores showed a slight improvement across all muscle groups despite the overall progression of structural abnormalities observed on MRI. This apparent discordance between structural progression and symptomatic improvement has been reported previously in KOA and likely reflects the well-recognized imperfect relationship between structural joint damage and patient-reported symptoms. In addition, regression to the mean, treatment uptake during follow-up, and selective attrition may have contributed to the observed improvement in WOMAC scores despite worsening structural disease.

The decline in CST performance observed in the muscle loss group further underscores the functional consequences of muscle decline. Physical performance tasks require both strength and coordination, and muscle loss may impair the ability to perform these activities, thereby exacerbating functional limitations. This finding is particularly concerning given the association between CST performance and broader measures of physical function and independence in KOA patients. Maintaining or increasing muscle volume appears critical for preserving physical performance, though interventions specifically designed to improve neuromuscular function may also play a role in mitigating declines.

The results of this study may have important implications for KOA management, particularly in the context of preventive and rehabilitative strategies. Although the present findings are observational and do not establish causality, they suggest that muscle loss, whether due to aging, inactivity, sarcopenia, or muscle wasting from weight [33,34], may represent a potentially modifiable risk factor associated with worsening KOA progression and impaired functional outcomes. Consequently, interventions aimed at preserving or increasing muscle volume, such as resistance training and physical activity programs, may be beneficial, consistent with current recommendations for KOA management [35]. Such interventions not only improve muscle strength and mass but also promote joint stability, reduce pain, and enhance mobility [36]. Moreover, addressing sarcopenia in older adults with KOA may be particularly beneficial, as this population is at increased risk for both muscle loss and joint damage. Additionally, dietary strategies, including protein-rich diets, may support muscle health and complement exercise-based interventions, particularly in populations at risk for muscle wasting [[37], [38], [39]]. However, whether interventions that increase muscle volume translate into slower structural progression of KOA remains to be established in prospective randomized intervention studies. A key strength of this study is the use of multi-slice-based volumetric muscle quantification, which provides a particularly robust approach for assessing longitudinal changes in muscle health [20] and extends prior single-slice cross-sectional area approaches [14] and may better reflect overall muscle morphology. In addition, the use of detailed MRI-based WORMS subscores allowed for a more granular assessment of structural changes, enabling the identification of tissue-specific associations, particularly for ligaments, tendons, and BMELL. However, MRI-based muscle assessment in KOA is increasingly moving toward multiparametric approaches that integrate for an example muscle volume and fat infiltration [20]. In this context, volumetric measures represent only one aspect of muscle health, and future studies should incorporate compositional MRI measures to provide a more comprehensive assessment.

Despite its strengths, this study has some limitations. The observational design precludes causal inference regarding the relationships between muscle volume changes and structural or functional outcomes. Furthermore, because of the observational design, the temporal relationship between changes in muscle volume and KOA progression cannot be established. Muscle decline may be both a cause and a consequence of KOA-related changes, and therefore reverse causation cannot be excluded. Additionally, while MRI-based WORMS scores provide detailed structural assessments, they do not account for dynamic biomechanical changes during movement that may contribute to joint instability and damage. Incorporating gait analysis or other biomechanical measures into future studies could provide a more comprehensive understanding of the interplay between muscle and joint health. Participants in the muscle loss group had higher baseline cartilage WORMS scores than the muscle gain group. Although the primary analyses were based on change scores rather than follow-up scores adjusted for baseline, we cannot exclude that baseline structural differences contributed to the observed progression patterns. Alternative analytical approaches, such as ANCOVA adjusting for baseline WORMS scores, may provide complementary insights [40,41]. Although baseline characteristics differed moderately between the participants that were excluded because of missing WORMS data and those that were included, with respect to BMI, whereas differences in physical activity were negligible. Nevertheless, some degree of selection bias due to missing outcome data cannot be excluded. Consequently, the present findings are most directly generalizable to OAI participants with available longitudinal WORMS assessments and should not automatically be extrapolated to the entire OAI cohort. The analysis was restricted to the right knee and right thigh. Consequently, our findings may not fully generalize to bilateral disease patterns or side-specific differences in muscle morphology and structural KOA.

In summary, this study underscores the critical role of muscle in mitigating KOA progression and improving functional outcomes. Muscle gain appears to protect against structural damage in ligaments, tendons, and BMELL while alleviating pain, whereas muscle loss exacerbates tendon damage and functional decline. These findings highlight the importance of preserving muscle volume in KOA management and suggest that targeted interventions to improve muscle health should be integral to comprehensive treatment strategies.

## Author contributions

The authors have made substantial contributions to the following sections:

- Conception and design (STU, ASG)
- Analysis and interpretations of the data (STU, NEL, CEM, MCN, ASG)
- Collection and assembly of data (STU, FL, GH, SM, TML, ASG)
- Drafting of the article (STU)
- Statistical expertise (GBJ, MCN, CEM)
- Critical revision of the article for important intellectual content (STU, FL, GH, GBJ, SM, MCN, CEM, NEL, TML, ASG)
- Final approval of the article (STU, FL, GH, GBJ, SM, MCN, CEM, NEL, TML, ASG)

## Role of the funding source

Research reported in this publication was supported by the 10.13039/100000069National Institute of Arthritis and Musculoskeletal and Skin Diseases of the 10.13039/100000002National Institutes of Health under Award Number P30AR075055. The content is solely the responsibility of the authors and does not necessarily represent the official views of the National Institutes of Health.

## Declaration of generative AI and AI-assisted technologies in the writing process

During the preparation of this work, the authors used GPT-4o by OpenAI to improve grammar, sentence structure, and enhance the readability of the manuscript. After using this tool, the authors reviewed and edited the content as needed and take full responsibility for the content of the publication.

## Competing interests

The authors declare no competing financial or personal interests related to this work. Given his role as Executive Guest Editor, Thomas M. Link had no involvement in the peer-review of this article and has no access to information regarding its peer-review. Full responsibility for the editorial process for this article was delegated to another journal editor.
