# Supplementary material for: Thigh muscle volume change is associated with longitudinal structural and functional knee osteoarthritis progression - Data from the osteoarthritis initiative
Source: Osteoarthr Cartil Open. 2026 Aug 13;8(3):100858. doi: 10.1016/j.ocarto.2026.100858 (PMC13499110; doi:10.1016/j.ocarto.2026.100858)
Supplement: Supplementary file 1 [file mmc1.docx]

| **Characteristic** | **Complete WORMS**  **(n = 1,715)** | **Missing WORMS**  **(n = 583)** | **P value** | **SMD** |
| --- | --- | --- | --- | --- |
| **Age, years** | 60.0 ± 8.9 | 61.3 ± 9.3 | 0.002 | 0.147 |
| **BMI, kg/m²** | 28.8 ± 4.3 | 26.6 ± 5.0 | <0.001 | 0.490 |
| **Sex** |  |  | <0.001 | 0.184 |
| Male | 784 (45.7) | 214 (36.7) |  |  |
| Female | 931 (54.3) | 369 (63.3) |  |  |
| **Race** |  |  | <0.001 | 0.271 |
| • Other Non-white | 20 (1.2) | 8 (1.4) |  |  |
| • White or Caucasian | 1397 (81.6) | 520 (89.2) |  |  |
| • Black or African American | 285 (16.6) | 47 (8.1) |  |  |
| • Asian | 11 (0.6) | 8 (1.4) |  |  |
| **PASE score** | 167.6 ± 82.5 | 164.7 ± 78.3 | 0.448 | 0.037 |

**Supplementary Table 1:** Comparison of baseline characteristics between participants with and without complete WORMS data. Continuous variables are presented as mean ± standard deviation (SD), and categorical variables as n (%). Between-group comparisons were performed using Welch's t-test for continuous variables and Pearson's χ² test for categorical variables. Standardized mean differences (SMDs) are provided to quantify the magnitude of between-group differences.
