# Supplementary material for: Thigh muscle volume change is associated with longitudinal structural and functional knee osteoarthritis progression - Data from the osteoarthritis initiative
Source: Osteoarthr Cartil Open. 2026 Aug 13;8(3):100858. doi: 10.1016/j.ocarto.2026.100858 (PMC13499110; doi:10.1016/j.ocarto.2026.100858)
Supplement: Supplementary file 2 [file mmc2.docx]

| **Outcome** | **Comparison** | **β (95% CI)** | **p-value** | **adjusted p-value*** |
| --- | --- | --- | --- | --- |
| **Primary outcomes** |  |  |  |  |
| WORMS Cartilage | Muscle gain vs. stable | -0.05 (-0.37, 0.27) | 0.75 | 0.75 |
|  | Muscle loss vs. stable | 0.15 (-0.17, 0.48) | 0.35 | — |
| WORMS Ligament | Muscle gain vs. stable | -0.12 (-0.22, -0.02) | 0.01 | 0.03 |
|  | Muscle loss vs. stable | -0.002 (-0.099, 0.095) | 0.97 | — |
| WORMS BMELL | Muscle gain vs. stable | -0.29 (-0.47, -0.10) | 0.002 | 0.009 |
|  | Muscle loss vs. stable | 0.03 (-0.16, 0.22) | 0.77 | — |
| WOMAC Pain | Muscle gain vs. stable | -0.36 (-0.68, -0.04) | 0.03 | 0.03 |
|  | Muscle loss vs. stable | -0.004 (-0.328, 0.320) | 0.98 | — |
| **Exploratory outcomes** |  |  |  |  |
| WORMS Meniscus | Muscle gain vs. stable | 0.01 (-0.21, 0.23) | 0.94 | — |
|  | Muscle loss vs. stable | 0.16 (-0.07, 0.38) | 0.17 | — |
| WORMS Tendon | Muscle gain vs. stable | -0.02 (-0.09, 0.04) | 0.48 | — |
|  | Muscle loss vs. stable | 0.08 (0.01, 0.15) | 0.02 | — |
| WOMAC Stiffness | Muscle gain vs. stable | -0.07 (-0.24, 0.11) | 0.47 | — |
|  | Muscle loss vs. stable | -0.003 (-0.178, 0.172) | 0.97 | — |
| WOMAC Disability | Muscle gain vs. stable | -0.88 (-1.83, 0.07) | 0.07 | — |
|  | Muscle loss vs. stable | -0.168 (-1.121, 0.785) | 0.729 | — |

**Supplementary Table 2:** Multivariable regression analyses of the association between muscle group and longitudinal changes in structural and symptomatic outcomes. BMELL = bone marrow edema-like lesions; CI = confidence interval; KOA = knee osteoarthritis; WORMS = Whole-Organ Magnetic Resonance Imaging Score. *Benjamini–Hochberg false discovery rate adjustment was applied only to the prespecified primary outcomes (WORMS BMELL, cartilage, ligament, and WOMAC pain). Exploratory outcomes were not adjusted for multiplicity and should be interpreted as hypothesis-generating.
