# Supplementary material for: Thigh muscle volume change is associated with longitudinal structural and functional knee osteoarthritis progression - Data from the osteoarthritis initiative
Source: Osteoarthr Cartil Open. 2026 Aug 13;8(3):100858. doi: 10.1016/j.ocarto.2026.100858 (PMC13499110; doi:10.1016/j.ocarto.2026.100858)
Supplement: Supplementary file 3 [file mmc3.docx]

| **Outcome** | **Comparison** | **Raw p-value** | **adjusted p-value** |
| --- | --- | --- | --- |
| WORMS BMELL | Muscle gain vs. stable muscle | 0.002 | **0.008** |
| WORMS Ligament | Muscle gain vs. stable muscle | 0.01 | **0.02** |
| WORMS Cartilage | Muscle gain vs. stable muscle | 0.75 | **0.75** |
| WOMAC Pain | Muscle gain vs. stable muscle | 0.03 | **0.04** |

**Supplementary Table 3:** Benjamini–Hochberg false discovery rate adjustment for prespecified primary outcomes. P-values were adjusted using the Benjamini–Hochberg false discovery rate procedure across the four prespecified primary outcomes.
