# Supplementary material for: Thigh muscle volume change is associated with longitudinal structural and functional knee osteoarthritis progression - Data from the osteoarthritis initiative
Source: Osteoarthr Cartil Open. 2026 Aug 13;8(3):100858. doi: 10.1016/j.ocarto.2026.100858 (PMC13499110; doi:10.1016/j.ocarto.2026.100858)
Supplement: Supplementary file 4 [file mmc4.docx]

| **Outcome** | **β (95% CI)** | **p-value** |
| --- | --- | --- |
| Δ WORMS Cartilage | -0.012 (-0.027, 0.004) | 0.144 |
| Δ WORMS Meniscus | -0.009 (-0.019, 0.002) | 0.120 |
| Δ WORMS Ligament | -0.003 (-0.008, 0.001) | 0.180 |
| Δ WORMS Tendon | -0.003 (-0.006, 0.0003) | 0.080 |
| Δ WORMS BMELL | -0.009 (-0.018, 0.0005) | 0.064 |

**Supplementary Table 4:** Multivariable linear regression models adjusted for age, sex, race, and baseline BMI. Percentage change in total muscle volume was analyzed as a continuous independent variable. β represents the regression coefficient for a one-unit (1%) increase in total muscle volume. Positive values of the outcome indicate greater progression of structural knee abnormalities over 48 months.
