# Supplementary material for: Thigh muscle volume change is associated with longitudinal structural and functional knee osteoarthritis progression - Data from the osteoarthritis initiative
Source: Osteoarthr Cartil Open. 2026 Aug 13;8(3):100858. doi: 10.1016/j.ocarto.2026.100858 (PMC13499110; doi:10.1016/j.ocarto.2026.100858)
Supplement: Supplementary file 5 [file mmc5.docx]

| **Outcome** | **Comparison** | **Original β (95% CI)** | **Original p** | **PASE-adjusted β (95% CI)** | **PASE-adjusted p** |
| --- | --- | --- | --- | --- | --- |
| Cartilage | Muscle gain vs. stable | -0.05 (-0.37, 0.27) | 0.751 | -0.06 (-0.38, 0.27) | 0.737 |
| Cartilage | Muscle loss vs. stable | 0.15 (-0.17, 0.48) | 0.353 | 0.17 (-0.15, 0.50) | 0.301 |
| Meniscus | Muscle gain vs. stable | 0.01 (-0.21, 0.23) | 0.937 | -0.01 (-0.23, 0.22) | 0.948 |
| Meniscus | Muscle loss vs. stable | 0.16 (-0.07, 0.38) | 0.168 | 0.16 (-0.07, 0.38) | 0.167 |
| Ligament | Muscle gain vs. stable | -0.12 (-0.22, -0.02) | 0.014 | -0.12 (-0.21, -0.02) | 0.019 |
| Ligament | Muscle loss vs. stable | -0.00 (-0.10, 0.10) | 0.966 | -0.00 (-0.10, 0.10) | 0.975 |
| Tendon | Muscle gain vs. stable | -0.02 (-0.09, 0.04) | 0.484 | -0.02 (-0.09, 0.04) | 0.496 |
| Tendon | Muscle loss vs. stable | 0.08 (0.01, 0.15) | 0.022 | 0.08 (0.01, 0.15) | 0.023 |
| BMELL | Muscle gain vs. stable | -0.29 (-0.48, -0.11) | 0.002 | -0.30 (-0.49, -0.11) | 0.002 |
| BMELL | Muscle loss vs. stable | 0.03 (-0.16, 0.22) | 0.767 | 0.03 (-0.15, 0.22) | 0.725 |

**Supplementary Table 5:** Sensitivity analysis of multivariable regression models additionally adjusted for baseline physical activity (PASE score).
