# Supplementary material for: Thigh muscle volume change is associated with longitudinal structural and functional knee osteoarthritis progression - Data from the osteoarthritis initiative
Source: Osteoarthr Cartil Open. 2026 Aug 13;8(3):100858. doi: 10.1016/j.ocarto.2026.100858 (PMC13499110; doi:10.1016/j.ocarto.2026.100858)
Supplement: Supplementary file 6 [file mmc6.docx]

| **Outcome** | **Comparison** | **Primary analysis (no outliers) β (95% CI)** | **P value** | **Sensitivity analysis (including outliers) β (95% CI)** | **P value** |
| --- | --- | --- | --- | --- | --- |
| **Primary outcomes** |  |  |  |  |  |
| WORMS Cartilage | Muscle gain vs. stable muscle | -0.05 (-0.37, 0.27) | 0.75 | 0.33 (−0.26, 0.93) | 0.27 |
|  | Muscle loss vs. stable muscle | 0.15 (-0.17, 0.49) | 0.35 | 0.47 (−0.13, 1.06) | 0.12 |
| WORMS Ligament | Muscle gain vs. stable muscle | -0.12 (-0.22, -0.02) | 0.01 | -0.12 (-0.22, -0.02) | 0.02 |
|  | Muscle loss vs. stable muscle | -0.002 (-0.099, 0.095) | 0.97 | 0.01 (-0.09, 0.11) | 0.83 |
| WORMS BMELL | Muscle gain vs. stable muscle | -0.29 (-0.48, -0.11) | 0.002 | -0.14 (-0.48, 0.21) | 0.43 |
|  | Muscle loss vs. stable muscle | 0.03 (-0.16, 0.22) | 0.77 | 0.09 (-0.25, 0.44) | 0.56 |

**Supplementary Table 6:** Sensitivity analysis including participants with extreme total muscle volume (TMV) changes. BMELL = bone marrow edema-like lesions; 95% CI = confidence interval; KOA = knee osteoarthritis; WORMS = Whole-Organ Magnetic Resonance Imaging Score.
